# Understanding menstrual factors associated with poor mental health among female secondary school students in Uganda: a cross-sectional analysis

**DOI:** 10.1186/s13034-024-00829-6

**Published:** 2024-10-14

**Authors:** Titus Ssesanga, Katherine A. Thomas, Kate Andrews Nelson, Evaline Oenen, Catherine Kansiime, Stephen Lagony, Jonathan R. Enomut, Yunia Mayanja, Helen A. Weiss

**Affiliations:** 1 MRC/UVRI and LSHTM Uganda Research Unit, Entebbe, Uganda; 2 MRC International Statistics and Epidemiology Group, London School of Hygiene & Tropical Medicine, London, UK; 3 Africa Research Excellence Fund (AREF), MRC Unit The Gambia at LSHTM, Fajara, The Gambia

**Keywords:** Menstrual health, Mental health, Socio-demographic factors, MENISCUS trial, Uganda, Adolescent girls, Strengths and difficulties questionnaire

## Abstract

**Background:**

There is growing global concern about poor mental health among adolescents in sub-Saharan Africa. In particular, adolescent girls face multiple challenges in managing menstruation which can impact both their health and wellbeing. In this study we address an evidence gap on the association of a broad range of menstrual-related factors with mental health problems amongst secondary school female adolescents in Uganda.

**Methods:**

We used baseline data from a cluster-randomised menstrual health intervention trial conducted in 60 secondary schools in two districts in Uganda. Baseline data were collected between March and July 2022, including socio-demographic characteristics, menstrual knowledge and attitudes, menstrual practices and self-efficacy, and mental health problems measured using the Strengths and Difficulties Questionnaire Total Difficulties score (SDQ-25). We used random-effects linear regression to estimate the adjusted mean difference (aMD) for the association between mental health problems (SDQ Total Difficulties Score) and menstrual-related factors, including the Menstrual Practice Needs Scale (MPNS) and the Self-Efficacy in Addressing Menstrual Needs scale (SAMNS)), accounting for school-level clustering and adjusting for prior confounders.

**Results:**

Among the 3841 female participants, there was strong and consistent evidence of associations between mental health problems and menstrual-related factors. Mental health problems were associated with poor knowledge about menstruation (aMD = 1.17, 95%CI 0.50, 1.84 <0.001), using disposable and reusable menstrual products compared to using just disposable products (aMD = 1.42, 95%CI 0.92, 1.92, *p* <0.001), and experiencing menstrual pain even when using an effective management method at last menstrual period (LMP) compared to those experiencing no pain (aMD = 1.60, 95%CI 1.19, 2.01, *p* <0.001). Mental health problems were also associated with greater unmet menstrual needs according to the MPNS (aMD = 4.40 95%CI 3.96, 4.84, *p* <0.001), and with lower menstrual self-efficacy measured by the SAMNS (aMD = 0.94 95% CI 0.51, 1.37, *p* <0.001).

**Conclusion:**

This study shows strong evidence that mental health problems reported by adolescent girls in Uganda are associated with poor menstrual health. The association between menstrual health and mental health provides evidence as to why menstrual health should be a public health priority.

**Trial registration:**

Trial registration: ISRCTN 45461276. Registered on 16 September 2021.

## Background

Mental health is defined as a state of well-being in which the individual realises his or her own abilities, can cope with the normal stresses of life, can work productively and fruitfully and is able to make contributions to the community [1]. Adolescents are particularly vulnerable to poor mental health due to physical, social and hormonal changes experienced during and after puberty [2–4]. There are gender imbalances in adolescent mental health, with girls being more likely to experience mental health disorders than boys [2, 5, 6].

There is a relative lack of research into the mental health of children and adolescents in low- and middle-income countries (LMICs) [7, 8]. A systematic review found a high prevalence of mental health disorders among adolescents in sub-Saharan Africa (SSA) compared with high income countries [9]. In this review, five studies involving 5390 adolescents in SSA reported on emotional and behavioural problems using the Strengths and Difficulties Questionnaire (SDQ). The median prevalence of mental health problems in two general population studies was 40.8% (IQR 31.2–41.4). Factors associated with poor mental health among adolescents in SSA include lower socioeconomic status, childhood trauma, orphanhood, gender-based violence, early marriage, and exclusion from education and employment [5, 9–11]. These factors coupled with the emotional and social changes occurring during puberty contribute to the burden of mental health problems among adolescents [12]. In addition, the COVID-19 pandemic has further exacerbated poor mental health among adolescents due to isolation, lack of social support and extended school closures [13].

For adolescent girls, menstruation can also cause challenges and put a strain on their mental health. Menstrual health is defined as a state of complete physical, mental and social well-being in relation to the menstrual cycle [14] and can impact girls’ physical health, employment and education [15–18]. The challenges faced by adolescent girls due to menstruation in LMICs are well documented, but there is limited research on the impact of broader dimensions of menstrual health on mental health using validated tools [19–22]. Research in this area has predominately looked at the association of mental health problems with specific menstrual issues (the effect of different phases of the menstrual cycle, dysmenorrhea or period poverty). In many settings, especially in LMICs, adolescent girls face broader challenges related to menstrual health including poor access to menstrual products and adequate water, sanitation and hygiene (WASH) facilities, and poor knowledge and attitudes around menstruation [23–25]. These factors can influence menstrual experience through reduced confidence and self-esteem, increased stress and anxiety which can result in poorer psychological health [22].

Recent research in SSA has highlighted an important association between puberty and menstruation-related stressors and mental health [11, 26]. In Tanzania, menstrual symptoms were found to be associated with depression and anxiety [26]. A study in Tanzania among adolescent girls and another the United States among college female students have reported that dysmenorrhea is a significant source of distress, affecting both educational achievements and psychological health, with many unable to afford menstrual health products, which can further impact their mental well-being [21, 27]. In addition, research in sub-Saharan Africa has found stigma and teasing from boys within a school environment can lead to negative emotional experiences for adolescent girls regarding menstruation [23, 28]. Despite this, research exploring how broader aspects of menstrual health is associated with mental health problems is limited.

This paper seeks to address the evidence gap on the associations of menstrual-related factors with secondary school adolescent girls’ mental health problems in Uganda. We aim to assess how sociodemographic and menstrual factors are associated with (i) poor overall mental health and (ii) poor internalized mental health, as measured by the Strengths and Difficulties Questionnaire Total Difficulties score (SDQ). Our hypothesis is that poor menstrual health is more likely to be associated with internalized mental health problems, such as depression, anxiety, and negative thoughts, rather than externalized mental health problems, characterized by hyperactivity and aggression which are more common in males [5, 29].

## Methods

### Study design and setting

This paper uses baseline data from the MENISCUS trial, a cluster-randomised trial which evaluated the impact of a multi-component menstrual health intervention in Ugandan secondary schools on girls’ education, health, and wellbeing [30]. The study was conducted in 60 schools (clusters) across two purposively-selected districts in central Uganda to include rural and peri-urban areas (44 schools in Wakiso District; 16 in Kalungu District). Secondary school education starts after 7 years of primary education and runs from Senior one (S1) to Senior 6 (S6).

### Participants

Schools were eligible for inclusion if they were mixed day/boarding or day schools, had an estimated enrolment of around 50–150 S1 students in Wakiso and 40–125 in Kalungu, were mixed sex secondary schools with S1-S4 classes and at least minimal WASH facilities (an improved water source and functional sex-specific sanitation facilities, usable, and accessible to female students) at the time of a rapid assessment eligibility survey in 2021 [31]. If schools were already enrolled in a menstrual-related program, they were not eligible for inclusion.

All female students in S2 in 2022 who were present at the time of the baseline survey, whose parent/guardian provided consent (if aged <18 years) and who assented/consented to the trial were eligible for inclusion in the trial. We enrolled students in S2 for practical reasons to allow parental consent to occur in S1, and to allow for trial follow-up in S3 whilst avoiding fieldwork during the examination year (in S4).

Our sample size of 3841 participants provides 90% power to detect a minimum mean difference of 0.90 for the SDQ total difficulties score to compare two equally sized tertiles of exposure (i.e. *N* = 1280 per exposure group), assuming a standard deviation of 5.6, Type 1 error of 0.05 and an intracluster correlation of 0.009. The standard deviation and intracluster correlation are those observed in the dataset overall.

### Data collection

Following informed assent, participants self-completed a baseline survey using Open Data Kit (ODK) software on a tablet at their school, between March-July 2022. The survey included questions on socio-demographic factors, knowledge on puberty and menstruation, participants menstrual experiences and mental health. Selected survey items were cognitively tested and refined iteratively in a sample of students to ensure understanding and that meanings of original items were adequately captured.

### Assessment of outcome and exposures

Mental health problems were assessed using the self-reported version of the SDQ Total Difficulties Score which has subscales including emotional symptoms (5 items), conduct problems (5 items), hyperactivity/inattention (5 items) and peer relationship problems (5 items) that measure internalizing and externalizing behaviour problems. The items are rated on a 3-point Likert scale from “not true” (0) to “certainly true” (2) to yield a total sum score in the range of 0–40. We did not include the prosocial sub-scale as it assesses resources rather than problems and is a conceptually different sub-scale [32]. A higher score indicates poorer mental health. Internalizing mental health problems were assessed by combining the items from the emotional symptoms and peer problems subscales [33].

The primary exposures were menstrual-related factors including years since menarche, knowledge of menstruation, knowledge of menstrual pain management strategies, type of menstrual product used at last menstrual period (LMP), use of effective pain management at LMP, the Menstrual Practice Needs Scale (MPNS-36) [34], and Self-efficacy in Addressing Menstrual Needs scale (SAMNS-26) [35]. The MPNS scale has previously been validated in a Ugandan adolescent population and the SAMNS has been validated in an adolescent population in Bangladesh [34, 35].

Menstrual pain management was measured as using at least one effective pain management strategy and no ineffective methods among girls reporting pain at their LMP(out of 9 possible strategies). Out of a total of 9 options, the 6 that were deemed effective methods were defined a-priori as use of painkillers, exercising, drinking lots of clean water, stretching, using a warm water bottle, and eating foods containing lots of water. Participants reporting 0–1 effective pain management methods were categorized as having poor knowledge of pain management methods, 2–3 items as medium and 4–6 as high. Menstrual knowledge was measured as the number of knowledge items answered correctly (out of 9). Participants answering 0–3 items correctly were categorized as having poor knowledge, those with 4–6 items as medium, and those with 7–9 items as good knowledge. Type of menstrual product was categorized into exclusive use of disposable products, exclusive use of reusable products or use of both. Experience of pain was categorized as ‘no pain at last menstrual period’, ‘pain without effective pain management’ and ‘pain with effective pain management’ if a participant recorded using any of the six effective pain management methods and no ineffective methods, among those that menstruated in the last 6 months.

The MPNS-36 measures the extent to which respondents’ menstrual management practices and environments were perceived to meet their needs during their LMP. Items ask about perceptions of comfort, satisfaction, adequacy, reliability as well as worries and concerns during their LMP. The mean total score is calculated as the mean response across all relevant items answered with items rated on a 4-point scale from ‘Never’ (0) to ‘All the time’ (3). A lower MPNS score corresponds to more unmet needs. The SAMNS-26 provides a measure of a participant’s confidence in her capabilities to address her menstrual needs (“menstrual self-efficacy”). Possible scores range from 0 to 100, 0% means “No, I cannot do it at all”, and 100% means “Yes, I am completely sure I can do it”, with a lower score reflecting poorer self-efficacy. We categorized the MPNS-36 and SAMNS-26 scores into tertiles.

Potential confounders measured were socio-demographic factors (age group, district, household size, type of student (day/boarder), socio-economic status (SES), education of primary caregiver) and age at menarche. SES was derived through principal components analysis using a household asset index and information on household characteristics.

### Statistical methods

**Fig. 1:**
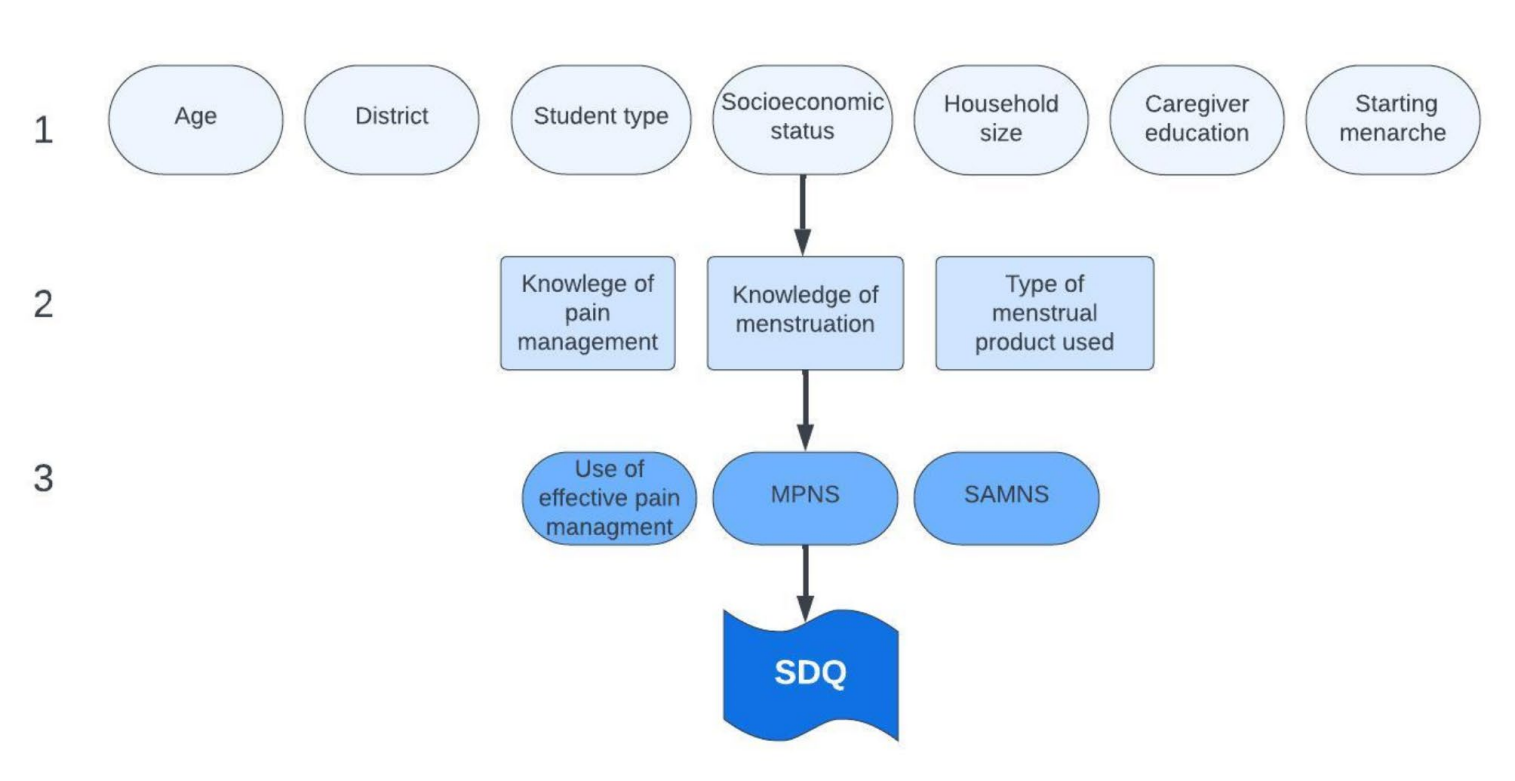
Conceptual framework for associations between menstrual related factors and SDQ score

Descriptive analyses were conducted comparing the mean SDQ score and standard deviation by categories of each exposure variable. We used linear regression models to estimate the adjusted mean differences (aMD) and 95% confidence intervals (CI) for the association of baseline SDQ total difficulties score with socio-demographic and menstrual-related variables at each level of the conceptual framework (Fig. 1). Minimally-adjusted models were fitted, adjusting for between-school clustering (random effects) and a-priori potential confounders (age group and district). A multivariable model was then fitted for each level of the framework, adjusted for other variables on that level and those on more distal levels. Multicollinearity for each model was assessed by observing standard errors (SE) of the main effects when additional variables were added, and removing variables that increased the SE substantially when included.

## Results

A total of 4,281 female students were eligible to be included for the baseline survey, of whom 399 (9.3%) were not enrolled due to either their parents declining consent (*n* = 238, 5.6%), the student declining assent (*n* = 12, 0.28%), or not being present at the time of the baseline survey (*n* = 149, 3.5%). An additional 4 participants withdrew their consent from the study, and 37 participants were recruited after the baseline survey. This resulted in a total of 3,841 (89.7%) participants who completed the baseline survey (median of 59 participants per school IQR 43–75). The mean age of participants was 15.5 years, the majority were day students (55.3%) and lived in a house with 6–10 other people (59.8%). Based on UK cut offs for the total SDQ score, 68% of participants scored as ‘close to average’, 15% were categorized as ‘slightly raised’, 7% as ‘high’ and 10% ‘very high’.

Table 1 shows that older participants had a higher mean SDQ score than younger participants (13.16 vs. 11.66). Participants who had low knowledge of menstrual pain management and low knowledgeon menstruation also had a higher mean SDQ score than participants with better knowledge (Table 1). The mean SDQ score for participants experiencing pain without effective pain management was considerably higher than for participants that reported experiencing no menstrual pain (13.13 vs. 10.44). Notably, the mean SDQ score showed a linear trend with participants having a higher SDQ score among those with a lower MPNS and SAMNs score (i.e. poorer menstrual health). The mean SDQ score of all participants was 12.16 (SD = 5.6) and the internal consistency for the total difficulties score was adequate (Cronbach’s alpha = 0.7), and lower for the internalized sub-scale (0.6).

**Table 1:** Distribution of study population and linear regression of factors associated with total difficulties score (*n* = 3,841)

	Total (percentage)	Mean (SD)	Mean difference*	Adjusted mean difference**	p-value
SDQ total difficulties score					
***Level 1 variables***					
**Age**					
11–15 years old	1,919 (50.0)	11.66 (5.47)	0	0	
16–17 years old	1,799 (46.8)	12.62 (5.66)	0.96 (0.60, 1.33)	0.55 (0.14, 0.97)	0.02
≥ 18 years old	123 (3.2)	13.16 (6.14)	1.49 (0.47, 2.51)	0.91 (-0.16, 1.99)	
**District**					
Kalungu	859 (22.4)	12.01 (5.42)	0	0	
Wakiso	2,982 (77.6)	12.20 (5.65)	0.28 (-0.25, 0.80)	0.48 (-0.09, 1.03)	0.09
**Student type**					
Day	2,124 (55.3)	12.23 (5.56)	0	0	
Boarding	1,717 (44.7)	12.07 (5.66)	0.08 (-0.31, 0.46)	0.19 (-0.21, 0.58)	0.35
**Household size**					
< 5 people	1,179 (30.7)	12.33 (5.42)	0		
6–10 people	2,297 (59.8)	12.06 (5.66)	-0.25 (-0.65, 0.14)	-0.26 (-0.66, 0.13)	0.43
10 + people	365 (9.5)	12.22 (5.79)	-0.13 (-0.79, 0.53)	-0.17 (0.84, 0.49)	
**Socioeconomic status**					
Lowest	771 (20.1)	13.43 (5.65)	0	0	
Medium-low	784 (20.4)	12.30 (5.48)	-0.49 (-1.05, 0.70)	-0.44 (-1.02, 0.14)	
Medium	757 (19.7)	11.81 (5.62)	-0.10 (-0.67, 0.46)	0.07 (-0.52, 0.66)	< 0.001t
Medium-high	770 (20.0)	11.39 (5.41)	0.34 (-0.22, 0.91)	0.42 (-0.18, 1.02)	
Highest	759 (19.8)	11.84 (5.64)	1.45 (0.87, 2.03)	1.55 (0.93, 2.17)	
**Caregiver education**					
More than secondary	1,007 (26.2)	11.95 (5.81)	0	0	
Secondary	1,270 (33.1)	12.13 (5.48)	0.02 (-0.45, 0.48)	-0.21 (-0.70, 0.27)	0.13
Primary or less	904 (23.5)	12.64 (5.79)	0.47 (-0.05, 0.98)	0.11 (-0.44, 0.65)	
Don’t know	660 (17.2)	11.87 (5.22)	-0.25 (-0.80, 0.30)	-0.51 (-1.08, 0.06)	
**Started menstruating**					
No	106 (2.7)	12.10 (5.45)			
Yes	3,705 (96.5)	12.15 (5.61)			
Don’t know	30 (0.8)	12.80 (5.77)			
**Years since starting menarche**					
< 2 years	937 (25.3)	11.71 (5.66)	0	0	
2 years	1,281 (34.6)	11.85 (5.46)	-0.59 (-1.03, -0.15)	-0.60 (-1.04, -0.17)	0.02
> 2 years	1,487 (40.1)	12.69 (5.66)	-0.59 (-1.10, -0.09)	-0.57 (-1.07, -0.06)	
***Level 2 variables***					
**Knowledge of menstrual pain management**					
Low 0/1	1,823 (47.5)	12.40 (5.69)	0	0	
Medium 2/3	1,355 (35.3)	11.91 (5.55)	-0.20 (-0.72, 0.32)	-0.23 (-0.75, 0.30)	0.02
High 4/6	663 (17.2)	11.99 (5.47)	0.29 (-0.21, 0.79)	0.33 (-0.18, 0.84)	
**Knowledge of menstruation**					
Low 0/3	559 (14.6)	12.97 (5.62)	0	0	
Medium 4/6	2,697 (70.2)	12.10 (5.59)	0.38 (-0.12, 0.88)	0.24 (-0.26, 0.75)	< 0.001t
High 7/9	585 (15.2)	11.63 (5.59)	1.24 (0.59, 1.89)	1.17 (0.50, 1.84)	
**Menstrual product used**					
Reusable only	638 (17.2)	13.15 (5.97)	0	0	
Disposable only	2,460 (66.5)	11.68 (5.37)	1.34 (0.85, 1.83)	1.14 (0.65, 1.64)	< 0.001
Both reusable & disposable	603 (16.3)	13.05 (5.92)	1.36 (0.87, 1.86)	1.42 (0.92, 1.92)	
***Level 3 variables***					
**Experience of menstrual pain**					
No pain	987 (26.7)	10.44 (5.25)	0	0	
Pain with effective pain management	1,709 (46.1)	12.56 (5.51)	2.02 (1.59, 2.45)	1.60 (1.19, 2.01)	< 0.001t
Pain with no effective pain management	1,009 (27.2)	13.13 (5.76)	2.60 (2.12, 3.09)	1.81 (1.35, 2.27)	
**Menstrual practice needs scale**					
Low	1,232 (33.5)	14.75 (5.54)	0	0	
Medium	1,214 (33.1)	12.05 (5.21)	2.40 (1.99, 2.81)	2.06 (1.64, 2.48)	< 0.001t
High	1,226 (33.4)	9.61 (4.81)	5.07 (4.66, 5.49)	4.40 (3.96, 4.84)	
**Self-efficacy of menstruation**					
Low	1,243 (33.6)	13.37 (5.60)	0	0	
Medium	1,232 (33.2)	12.17 (5.55)	1.22 (0.78, 1.65)	0.66 (0.25, 1.07)	< 0.001t
High	1,230 (33.2)	10.90 (5.40)	2.44 (2.00, 2.87)	0.94 (0.51, 1.37)	

*Adjusted for a priori confounders age and district

**Level 1 variables are adjusted for each other; level 2 variables are adjusted for level 1 & 2 variables and level 3 variables are adjusted for all other variables

t Test for trend

Participants over the age of 18 had on average a higher mean SDQ score than those under the age of 15 years old (adjusted mean difference (aMD) = 0.91; 95% CI: -0.16, 1.99; *p* = 0.02). There was a strong linear association between SES and SDQ score with those of lowest SES having on average a higher SDQ score than those in the highest SES category (aMD = 1.45; 95%CI: 0.87, 2.05; *p* < 0.001). There was strong evidence that SDQ scores were lower among those with fewer years since the start of menarche (aMD=-0.57; 95%CI: -1.07, 0.06; *p* = 0.02 for < 2 vs. > 2 years duration), poor levels of menstrual knowledge (aMD = 1.17; 95%CI: 0.50, 1.84; *p* < 0.001) and those that experienced menstrual pain even with effective pain management (aMD = 1.60; 95%CI 1.19, 2.01; *p* < 0.001) (Table 1). There was strong evidence that SDQ scores were higher among participants with lower (poorer) MPNS scores (aMD = 4.40; 95%CI: 3.96, 4.82 for low vs. high scores; *p* < 0.001) and to a lesser extent with poorer SAMNS scores (aMD = 0.94 95%CI 0.51–1.37 for low vs. high scores; *p* < 0.001).

Similar results were seen for the internalised SDQ subscale (Table 2). Higher internalised SDQ scores were associated with older age (aMD = 0.49; 95%CI: -0.19, 1.17 *p* = 0.05) and lower SES (aMD = 1.07; 95%CI: 0.68, 1.47 *p* < 0.001). Poor menstrual knowledge was also linearly associated with poor mental health (aMD = 0.44; 95%CI 0.16, 0.72 for low vs. high scores *p* = 0.002). Participants who used reusable menstrual products (aMD = 0.49; 95%CI: 0.17, 0.81 *p* < 0.002) and experienced menstrual pain even with effective pain management (aMD = 1.26; 95%CI 0.99, 1.52 *p* < 0.001) had higher internalised SDQ scores. Similarly to the total SDQ score, both a higher MPNS and SAMNS displayed a linear association with a lower internalised SDQ score.

**Table 2:** Linear regression of factors associated with internalised SDQ subscale

Level 1 variables	Mean difference*	Adjusted mean difference**	*p*-valuet
**Age**			
11–15	0	0	0.05
16–17	0.58 (0.35, 0.81)	0.30 (0.04, 0.56)	
≥ 18 years old	0.89 (0.24, 1.53)	0.49 (-0.19, 1.17)	
**District**			
Kalungu	0	0	
Wakiso	0.27 (-0.47, 0.60)	0.41 (0.06–0.76)	0.02
**Student type**			
Day	0		
Boarding	0.03	0.13	0.29
**SES**			
Highest	0	0	
Medium-high	-0.06 (-0.42, 0.29)	-0.06 (-0.43, 0.31)	
Medium	0.27 (-0.09, 0.63)	0.36 (-0.01, 0.74)	< 0.001t
Medium-low	0.41 (0.05, 0.77)	0.45 (0.07, 0.83)	
Lowest	1.04 (0.67, 1.40)	1.07 (0.68, 1.47)	
**Household size**			
< 5 people	0		
6–10 people	-0.16 (-0.41, 0.09)	-0.17 (-0.42, 0.09)	0.41
> 10 people	-0.03 (-0.44, 0.39)	-0.05 (-0.47, 0.37)	
**Caregiver education**			
More than secondary	0	0	
Secondary	0.04 (-0.25, 0.34)	-0.13 (-0.44, 0.17)	0.12
Primary or less	0.40 (0.07, 0.72)	0.17 (-0.18, 0.51)	
Don’t know	-0.05 (-0.40, 0.30)	-0.24 (-0.60, 0.12)	
**Years since started menarche**			
> 2 years	0	0	0.02
2 years	-0.36 (-0.64, -0.08)	-0.37 (-0.65, -0.09)	
< 2 years	-0.38 (-0.71, -0.06)	-0.36 (-0.68, -0.04)	
***Level 2 variables***			
**Knowledge of pain management**			
Good 4/6	0	0	
Medium 2/3	-0.10 (-0.43, 0.23)	-0.11 (-0.45, 0.22)	0.06
Poor 0/1	0.19 (-0.13, 0.50)	0.20 (-0.13, 0.52)	
**Knowledge of menstruation**			
Good 7/9	0	0	
Medium 4/6	0.20 (-0.11, 0.52)	0.13 (-0.19, 0.45)	0.01t
Poor 0/3	0.61 (0.20, 1.03)	0.56 (0.14, 0.99)	
**Type of menstrual product**			
Disposable only	0	0	< 0.001
Reusable only	0.64 (0.33, 0.95)	0.46 (0.15, 0.78)	
Both disposable and reusable	0.62 (0.31, 0.94)	0.62 (0.30, 0.94)	
***Level 3 variables***			
**Use of effective pain management**			
No pain	0	0	< 0.001t
Pain with effective pain management	1.48 (1.21, 1.75)	1.23 (0.96, 1.49)	
Pain without effective pain management	1.72 (1.42, 2.02)	1.28 (0.98, 1.57)	
**MPNS**			
High (2.36- 3)	0	0	
Medium (1.87 < 2.36)	1.39 (1.12, 1.65)	1.18 (0.91, 1.45)	< 0.001t
Low (0 < 1.87)	2.85 (2.59, 3.12)	2.45 (2.16, 2.73)	
**SAMNS**			
High (70–100)	0	0	
Medium (52.4 < 70)	0.68 (0.40, 0.96)	0.36 (0.10, 0.63)	0.003t
Low (0 < 52.4)	1.26 (0.99, 1.54)	0.41 (0.13, 0.69)	

*Adjusted for a priori confounders age and district

**Level 1 variables are adjusted for each other; level 2 variables are adjusted for level 1 & 2 variables and level 3 variables are adjusted for all other variables

t Test for trend

## Discussion

This is the first study to examine the association of mental health problems with a broad range of menstrual-related factors. We found strong and consistent evidence of associations between poor menstrual health and poor mental health among Ugandan adolescent schoolgirls, including menstrual knowledge, pain, product use and most notably with the menstrual practice needs and self-efficacy scales. The results showed a similar pattern for the internalised SDQ sub scale.

Our key novel finding is the strong evidence of association between mental health problems and the MPNS and SAMNS scales among school-going adolescents. Increased mental health problems were associated with lower self-efficacy to manage menstruation, and with lower perceived ability to meet their menstrual practice needs. This suggests that even among girls with a similar physical and economic environment, poorer menstrual self-efficacy is associated with increased stress and anxiety. These results align with a qualitative systematic review among women and girls in LMICs that found poor confidence to manage menstruation impacts broader mental health such as anxiety and depression [22].

As found in other populations, older adolescents (aged *≥* 16 years) were more likely to experience mental health problems than younger adolescents [11]. This is possibly due to stress, educational pressures and peer relations which are heightened in the later adolescent years [2–4]. In the context of our study, conducted among S2 students, older participants would have started school later or been held back. Being older than one’s peers in school could itself be associated with poorer mental health, particularly as these reasons for being held back could be related to disruptions due to Covid-19.

Our findings also highlight the association between menstrual pain and poor mental health. This is consistent with previous research highlighting that primary dysmenorrhea affects the psychological well-being of individuals, potentially contributing to increased levels of stress, anxiety, and overall diminished quality of life [27, 36]. We found an association between menstrual pain and SDQ score but did not observe any notable difference in SDQ score between those who reported effectively managing their pain compared to those who did not effectively manage their pain. This may reflect limitations in our definition of “effective” pain management strategies, i.e. the strategies deemed to be effective by our definition may not align with what strategies girls found effective to manage their own pain.

The results show that reporting any use of reusable menstrual products compared to exclusive use of disposable products was associated with increased mental health problems. This could be due to the convenience in using disposable products compared to more complex management of reusable menstrual products, which require access to water and a private space to dry and hang the products [37, 38]. It is also plausible this finding is due to chance or residual confounding from variables such as WASH infrastructure.

A strength of the study is the use of validated tools to measure constructs of menstrual health that focus on adolescents’ perceptions and confidence in managing their menstruation. Whilst studies typically focus on the physical and social environment to assess menstrual management, the MPNS and SAMNS scale seek to understand how a participant feels about their menstrual practices providing a more comprehensive picture of adolescents’ menstrual experiences [22, 35]. Other notable strengths include the randomized selection of schools for inclusion in the study, as well as consistently high response rates at the individual level. Additionally, the schools included exhibited diversity in various aspects such as academic achievement, size, composition of day and boarding students, and location (rural or urban). To minimize observer bias, students self-completed the survey on tablets, and participants were assured of the anonymity of their data, fostering a comfortable environment for honest responses.

Limitations of the research include that the findings from this population of Ugandan adolescent schoolgirls may not be generalizable to adolescents in other school-based settings. As students are required to pay fees to attend school within Uganda, the findings may not be representative of all adolescent girls. The cross-sectional design of the study prevents establishing causation, and reverse-causality is possible as poor mental health might contribute to poorer menstrual experiences. Additionally, reliance on self-reported data, particularly for mental health and menstrual experiences could introduce potential biases related to social desirability and recall which may affect the reliability and accuracy of the findings. It could also be possible that there is residual confounding which alters the association between menstrual health and mental health by variables that were not collected in this survey e.g. information on history of prior mental health, their home environment, gender based violence and early marriage [5, 10, 11].

Despite the utility of the SDQ in identifying behavioral and emotional difficulties, it does have limitations, particularly in its lack of ability to diagnose specific mental health disorders like depression and anxiety [33]. In our study, we measured the SDQ as a continuous scale rather than categorizing children into distinct groups. This approach allows for a nuanced understanding of mental health variations and facilitates early intervention and support by capturing even small changes in SDQ scores as meaningful differences in mental health status [32, 39].

## Conclusions

Our findings highlight the association between poor menstrual health and mental health problems in Ugandan school-going adolescents. This supports findings from other settings and broadens the evidence for an association beyond dysmenorrhea and menstrual management to include a range of menstrual-related factors including unmet menstrual needs and poor menstrual self-efficacy. This association between girls’ perceptions and confidence in their menstruation is important to understand if we are to consider menstrual health more holistically beyond the physical and economic environment. Interventions to improve menstrual health should be considered alongside other interventions to improve mental health in adolescents.

## Data Availability

The data that support the results of this manuscript have been deposited in the LSHTM Data Compass repository here: https://doi.org/10.17037/DATA.00003865.

## Abbreviations

SDQ: Strengths and difficulties questionnaire
SES: Socio-economic status
MPNS: Menstrual practice needs scale
SAMNS: Self-efficacy in addressing menstrual needs scale
WASH: Water, sanitation and hygiene
